# Predictive models in SMA II natural history trajectories using machine learning: A proof of concept study

**DOI:** 10.1371/journal.pone.0267930

**Published:** 2022-05-05

**Authors:** Giorgia Coratti, Jacopo Lenkowicz, Stefano Patarnello, Consolato Gullì, Maria Carmela Pera, Carlotta Masciocchi, Riccardo Rinaldi, Valeria Lovato, Antonio Leone, Alfredo Cesario, Eugenio Mercuri

**Affiliations:** 1 Pediatric Neurology, Università Cattolica del Sacro Cuore, Rome, Italy; 2 Centro Clinico Nemo, Fondazione Policlinico Universitario Agostino Gemelli IRCCS, Rome, Italy; 3 Fondazione Policlinico Universitario A. Gemelli IRCCS, Università Cattolica del Sacro Cuore, Rome, Italy; 4 Department of Radiological and Hematological Sciences Fondazione, Policlinico Universitario A. Gemelli, IRCCS Università Cattolica del Sacro Cuore, Largo A. Gemelli, Rome, Italy; 5 Roche S.p.A., Monza, Monza e Brianza, Italy; 6 Open Innovation Manager, Scientific Directorate, Fondazione Policlinico Universitario A. Gemelli IRCCS, Università Cattolica del Sacro Cuore, Rome, Italy; UCL: University College London, UNITED KINGDOM

## Abstract

It is known from previous literature that type II Spinal Muscular Atrophy (SMA) patients generally, after the age of 5 years, presents a steep deterioration until puberty followed by a relative stability, as most abilities have been lost. Although it is possible to identify points of slope indicating early improvement, steep decline and relative stabilizations, there is still a lot of variability within each age group and it’s not always possible to predict individual trajectories of progression from age only. The aim of the study was to develop a predictive model based on machine learning using an XGBoost algorithm for regression and report, explore and quantify, in a single centre longitudinal natural history study, the influence of clinical variables on the 6/12-months Hammersmith Motor Functional Scale Expanded score prediction (HFMSE). This study represents the first approach to artificial intelligence and trained models for the prediction of individualized trajectories of HFMSE disease progression using individual characteristics of the patient. The application of this method to larger cohorts may allow to identify different classes of progression, a crucial information at the time of the new commercially available therapies.

## Introduction

Spinal muscular atrophy (SMA) is an autosomal recessive disorder characterized by loss of motor neurons with subsequent progressive muscle weakness and wasting [1]. Classically, SMA is described into subtypes (0-IV) based on age of onset and maximum function achieved, with type II patients achieving the ability to sit but not to walk independently [2, 3]. Several studies have reported natural history longitudinal data in type II SMA, mostly using disease specific outcome measures such as the Hammersmith Functional Motor Scale (HFMSE) or Revised Upper Limb Module (RULM) [4–9].

Type II patients generally present onset of clinical signs between 6 and 18 months of age, after they have achieved the ability to sit independently [2, 10]. In the first years after diagnosis, there may be some improvement in motor function that however is not constantly observed. Conversely, after the age of 5 years there is often a steep deterioration until puberty, with loss of several functional abilities, followed by a relative stability [5, 8, 9].

Both cross sectional and longitudinal studies have identified points of slope indicating early improvement, steep decline and relative stabilizations occurring at different ages in type II patients, there is still a lot of variability within each age group and it’s not always possible to predict individual trajectories of progression from age only [5, 8, 11]. In the last few years there has been an effort to identify predictors of progression in several diseases, including some neuromuscular disorder such as Duchenne muscular dystrophy [12–14]. These studies have suggested that the possibility to predict progression increases by combining different variables in a composite model. To our knowledge, the possibility to predict progression in SMA using a number of clinical variables, such as weight, scoliosis, ventilation or nutritional status has not been systematically explored.

The aim of the study was to develop a predictive model and report, explore and quantify, in a single center longitudinal natural history study, the influence of clinical variables on the 6/12-months HMFSE prediction.

## Material and methods

### Cohort selection and dataset definition

All patients included had a genetic diagnosis of SMA and a phenotype compatible with type II, i.e. onset between 6 and 18 months and independent standing and walking never achieved. All the patients older than 2.5 years with at least three assessments were included. Assessments performed at the time the patients were treated with investigational or approved disease modifying therapies such as nusinersen or risdiplam were not included. The final dataset was created by taking the complete cases record (i.e. no missing values), retrieved form medical records, of all the available variables: gender, *SMN2* copies, age at visit, age at symptom onset, anthropometric measures, Cobb values, vitamin D treatment, SMA specific surgeries (spinal or tendon surgeries), salbutamol treatment, acute hospitalizations, ventilatory and nutritional status. HFMSE total score and functional status (non sitters/sitters) were considered and included as an indicator of motor function. Height, weight and Cobb values were imputed for missing values via linear interpolation between visits with non-missing values.

### XGBoost algorithm

XGBoost is a popular and efficient open-source implementation of the gradient boosted trees algorithm. Gradient boosting is a supervised learning algorithm, which attempts to accurately predict a target variable by combining the estimates of a set of simpler, weaker models. When using gradient boosting for regression, the weak learners are regression trees, and each regression tree maps an input data point to one of its leafs that contains a continuous score. XGBoost minimizes a regularized (L1 and L2) objective function that combines a convex loss function (based on the difference between the predicted and target outputs) and a penalty term for model complexity (in other words, the regression tree functions). The training proceeds iteratively, adding new trees that predict the residuals or errors of prior trees that are then combined with previous trees to make the final prediction. It’s called gradient boosting because it uses a gradient descent algorithm to minimize the loss when adding new models. Advantages of using this algorithm include: the non-linearity it introduces in the association among predictor variables and outcome; the ensemble of weak learners approach helps to prevent overfitting by an appropriate tuning of the model’s hyperparameters; it has built-in regularization terms in the loss function which help reduce overfitting and improve generalization; finally, it is designed to be computationally efficient and to support parallel and distributed computing, which is useful to explore wider hyperparameters spaces, and eventually perform incremental training in a multicentric setting without in principle sharing the actual datasets.

No preliminary feature selection was performed, and all the available variables in the dataset were given as input to the training algorithm. The algorithm itself therefore assigns a higher or lower importance score to each variable so that, at the end of the training phase, the different variables are ideally ranked by their importance in an optimal way. The study was approved by the institutional review board (ethics committee) of the Fondazione Policlinico Agostino Gemelli (project code:GEN-SMA01, prot n°0019648/21). Written informed consent was obtained from all participants (or guardians of participants) in the study.

### Visit-by-visit analysis

**Fig 1:**
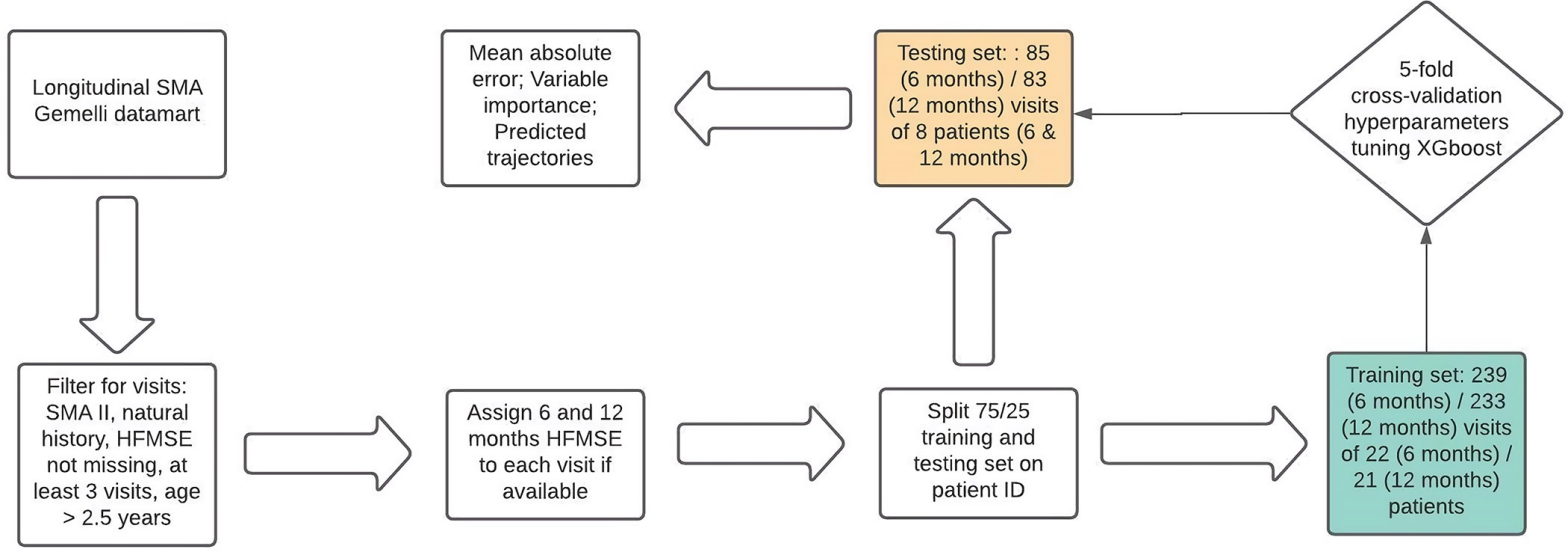
Workflow of the machine learning analysis for the model.

The model was trained to predict the HFMSE value after 6 or 12 months from a given visit, based on the actual visit variables. For each time-point, the visit data were linked to the HFMSE value closest to 6 or 12 months from the visit date, which becomes the outcome of the predictive model. Data were extracted from the Gemelli datamart for Spinal Muscular Atrophy, were filtered by the inclusion/exclusion criteria and were then included in the model. After the construction of the datamart, the data were split into a training set and testing set at a 75%/25% proportion. An XGBoost algorithm for regression was run in 5-fold cross-validation on the training set for hyperparameters optimization, for a total of 5400 different models. The best model was chosen according to lowest cross-validation Root Mean Squared Error (RMSE). The model was then applied on the testing set to measure the RMSE and the Mean Absolute Error (MAE). The complete workflow is depicted in Fig 1.

## Results

### 6-months prediction

Considering the 6-month interval and applying the filtering criteria, a total of 30 patients and 324 visits were included. Average number of visits per patient was 10.8, with a minimum of 3.0, a maximum of 23.0 and interquartile range 7.2–13.7. According to the machine learning model described above, 22 patients were assigned to the training set (239 visits), and 8 patients (85 visits) were assigned to the testing set. Of the patients included in the analysis, 2 patients (training set) were able to stand with support at truck. Table 1 shows the dataset characteristics summary subdivided by training and testing set.

**Table 1:** 6-months HMFSE dataset summary. Numerical values are reported as median and interquartile range.

	total	training set	testing set	p-value
patients	30	22	8	-
visits	324	239	85	-
visits per patient	11.0 (7.25–13.75)	11.0 (6.50–13.75)	11.0 (7.75–13.25)	0.90
gender male gender female	17	13	4	0.65
	13	9	4	
age at first visit (years)	3.31 (2.85–5.72)	3.70 (2.94–5.92)	2.93 (2.81–3.14)	0.03
HMFSE at first dataset visit	16.00 (9.50–21.75)	15.50 (8.75–22.0)	17.00 (11.25–19.50)	0.80
age symptoms onset	0.88 (0.59–1.00)	0.91 (0.58–1.00)	0.69 (0.59–1.00)	0.40

**Fig 2:**
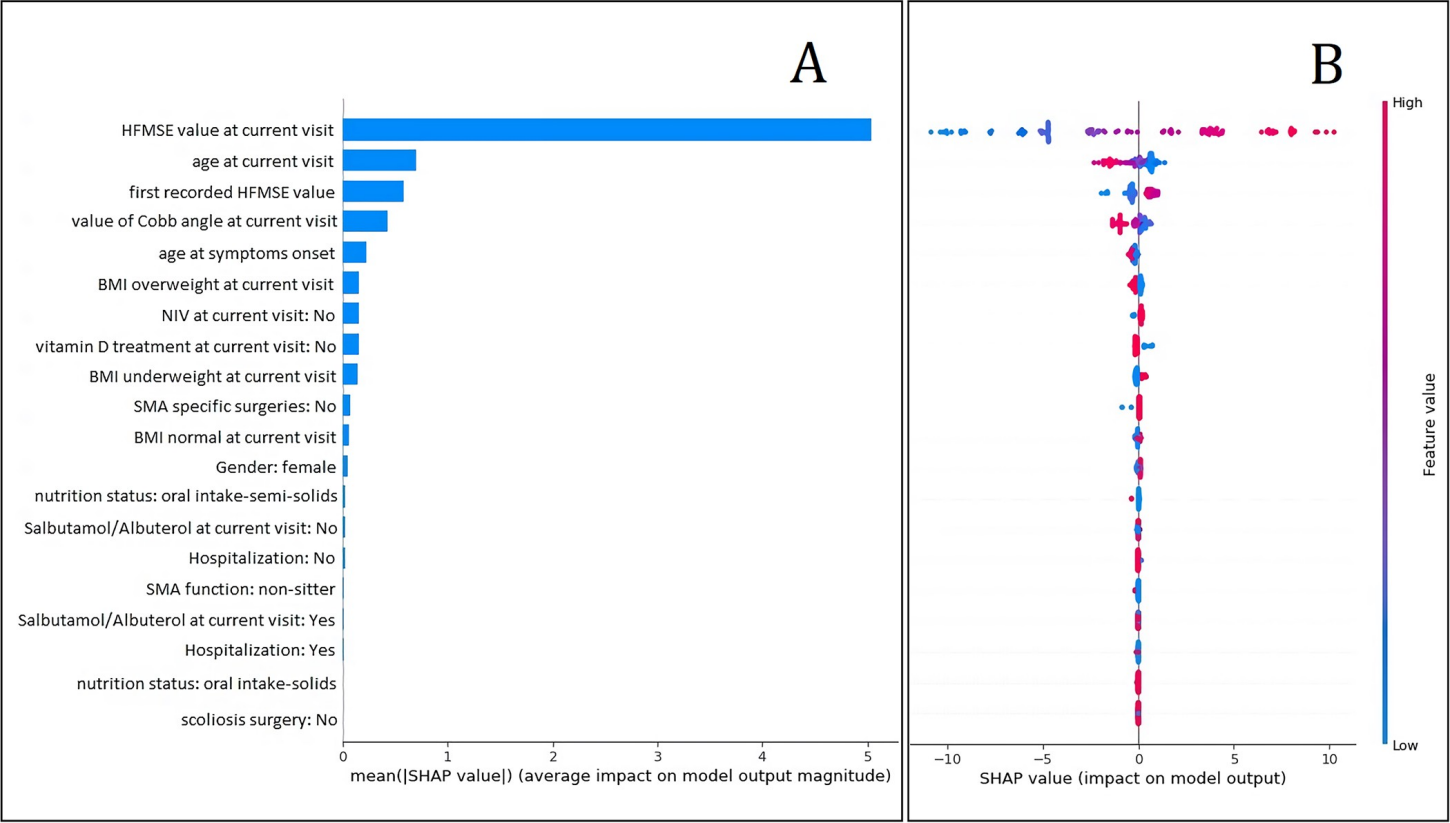
Top 20 features importance for 6 months model according to mean SHAP value (A) and SHAP value (B).

**Fig 3:**
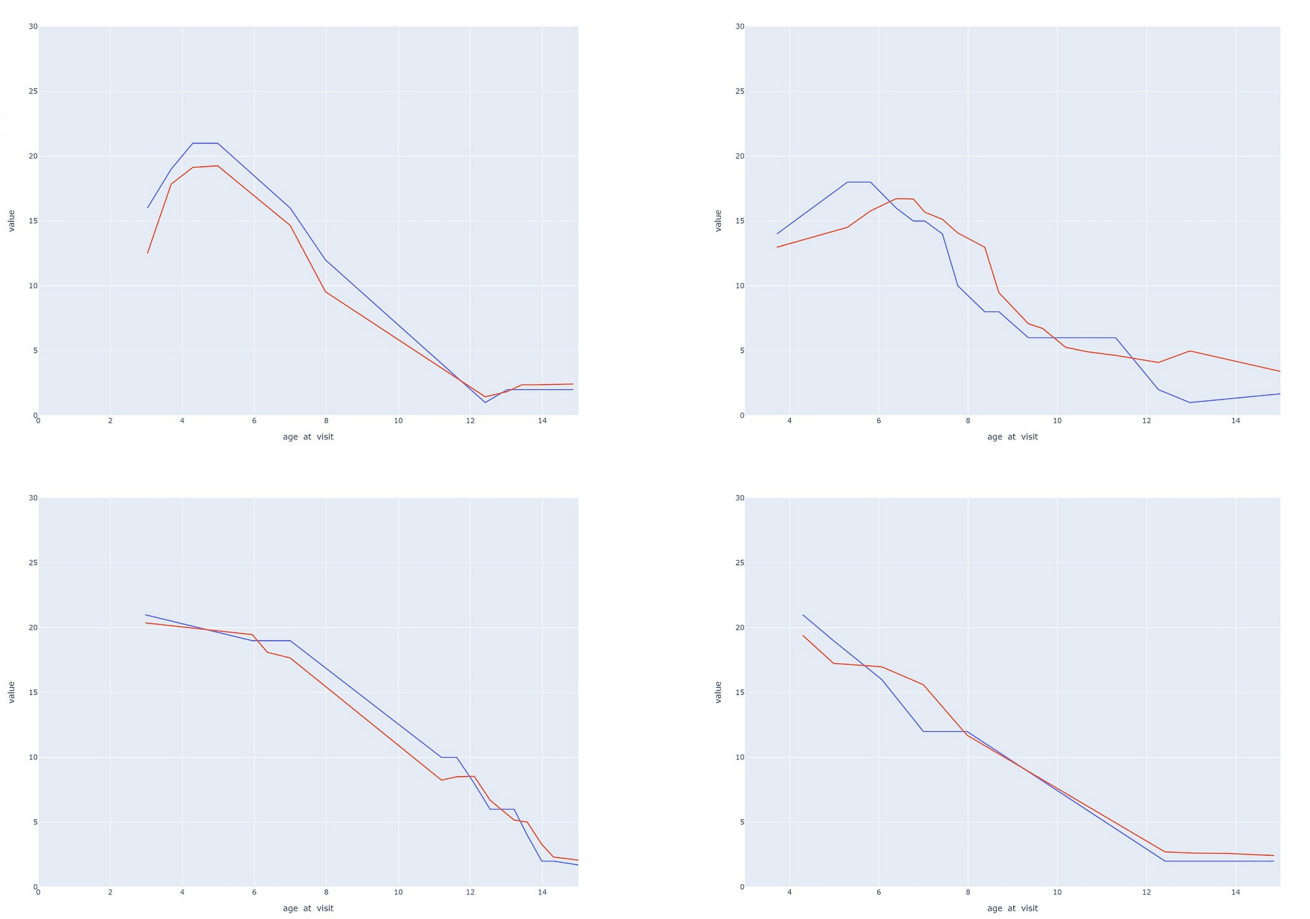
Trajectory predictions on the testing set for 4 patients. Key to figure: Blu line = actual HFMSE progression, Red line = model-predicted HFMSE progression.

The best XGBoost model showed a testing MAE of 1.86 HFMSE points. The five most important variable for the model according to the Shapley Additive Explanations (SHAP) framework are (Fig 2A): HFMSE value at given visit, age at current visit, first recorded HFMSE value, value of Cobb angle at current visit, age at symptoms onset. The prediction for 6-month value of HFMSE are influenced towards higher values by: a higher HFMSE value at current visit, a lower age at visit, a higher value of first recorded HFMSE and a lower Cobb angle (Fig 2B). Fig 3 shows four examples of visit-by-visit trajectory as predicted by the model compared to the actual values.

### 12-months prediction

Considering the 12-month interval and after applying the new filtering criterion, patients eligible for the analysis were 29, for a total of 316 visits. Average number of visits per patient was 10.9, with a minimum of 3.0, a maximum of 24.0 and interquartile range 7.0–14.0. Of these, 21 patients were assigned to the training set (233 visits), and 8 patients (83 visits) were assigned to the testing set.

Table 2 reports the dataset summary characteristics applying to the 12-months interval.

**Table 2:** 12-months HMFSE dataset summary. Numerical values are reported as median and interquartile range.

	total	training set	testing set	p-value
patients	29	21	8	-
visits	316	233	83	-
visits per patient	11.0 (7.0–14.0)	12.0 (7.0–15.0)	9.0 (7.75–12.25)	0.73
gender male gender female	16	10	6	0.18
	13	11	2	
age at first dataset visit (years)	3.45 (2.84–4.93)	2.97 (2.84–4.93)	3.69 (3.40–4.72)	0.50
HMFSE at first dataset visit	16.0 (11.0–22.0)	16.0 (11.0–22.0)	16.0 (11.25–20.0)	0.74
age symptoms onset	0.85 (0.58–1.00)	0.91 (0.67–1.00)	0.63 (0.58–0.81)	0.14

**Fig 4:**
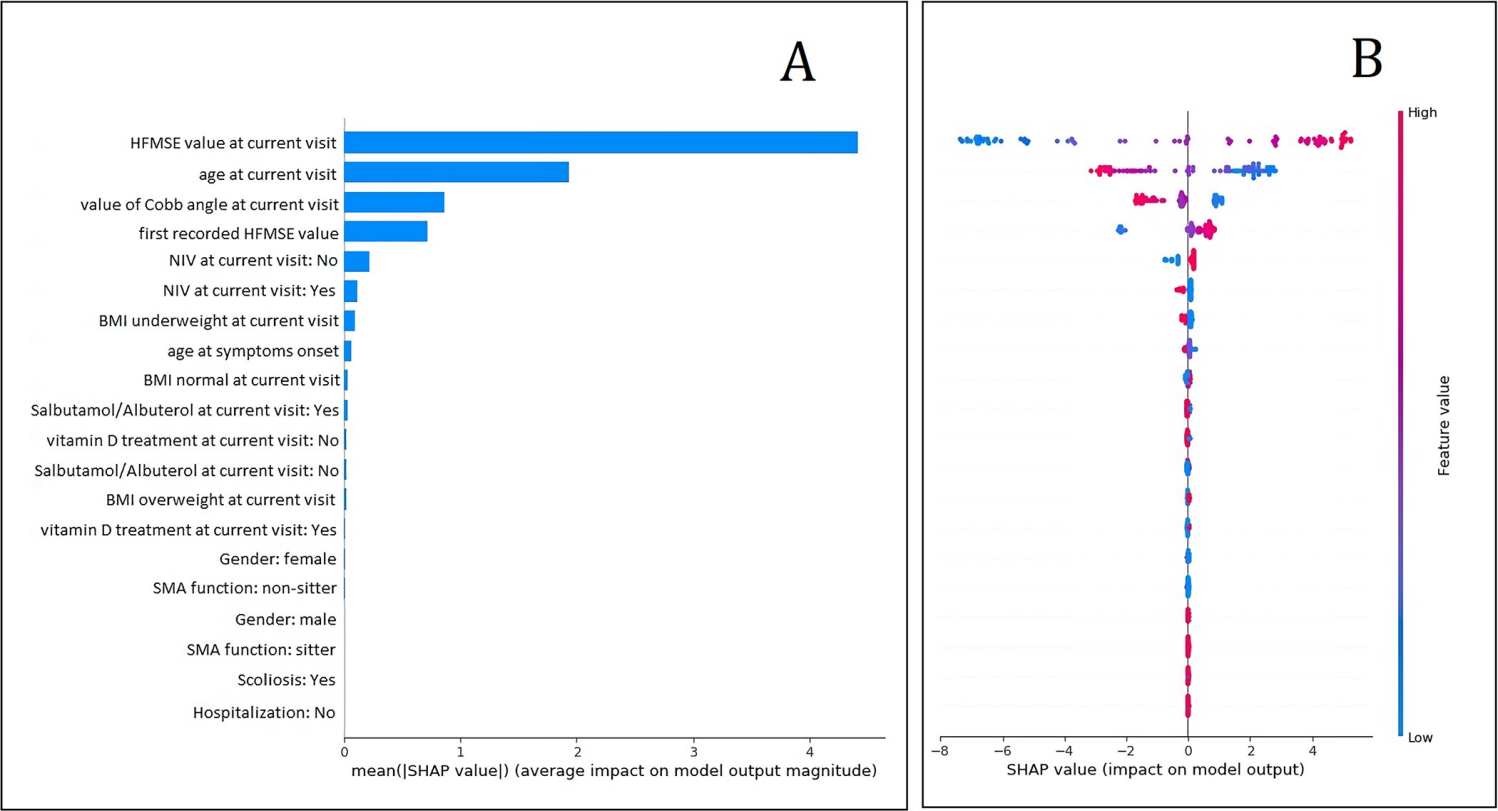
Top 20 features importance for 6 months model according to mean SHAP value (A) and SHAP value (B).

The best XGBoost model showed a testing set MAE of 1.97 HFMSE points. The five most important variable for the model according to the SHAP framework are: HFMSE value at current visit, age at current visit, Cobb angle, first recorded HFMSE value, Non-invasive ventilation at current visit (Fig 4A and 4B).

**Fig 5:**
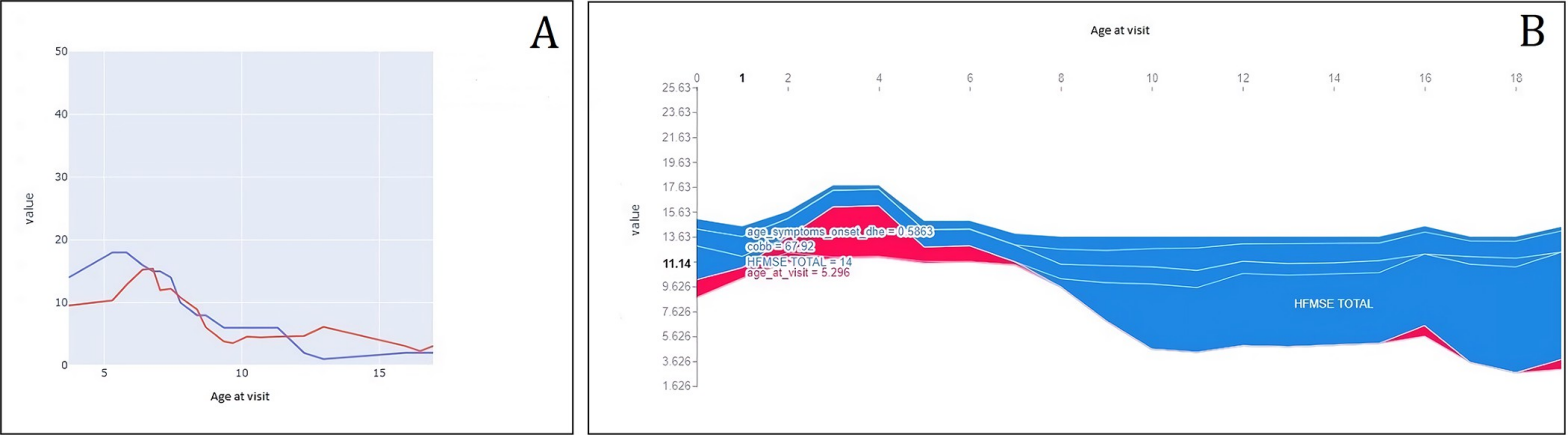
Prediction (A) and corresponding prediction by-prediction (B) for a sample testing patient. Key to Fig = Panel A: comparison between the actual 12 months trajectory (blue) of a testing set patient and the corresponding trajectory predicted by the 12-months model (red). Panel B: a focus on the predicted trajectory to show the variables contributing to the predictions along the trajectory, according to the SHAP framework. The red area represents positive contribution to the prediction, while the blue area represent negative contribution (e.g.: the first prediction (HFMSE equal to 11.14) relies on the positive contribution of age at current visit (5.3 years), and the negative contributions of age at symptoms onset (0.58 years), Cobb angle value (67.9), and previous HFMSE value).

A prediction for a sample testing patient and corresponding prediction-by-prediction SHAP values is reported in Fig 5 and the explanation in Fig 5.

## Discussion

Several international efforts have reported that in SMA type 2 patients motor function progression is not linear and that different slopes of progression can be identified using appropriate functional measures such as the HFMSE [5, 8, 9, 15–17] or other measures [7, 18–20]. In some of those studies, using multivariate/descriptive analysis per cohort or subgroup of patients, age appears to be an important predictor. Children younger than 5 years appear to have the highest chances of showing an improvement in HFMSE scores while those between 5 and 13 years are in contrast most susceptible to negative changes. Even when using age subgroups based on these cutoff points, there was still a high variability that could not be explained by multivariate analysis including other variables such as gender or *SMN2* copies.

The objective of this study was not to describe HFMSE disease progression by cohorts or pre-defined subgroups (e.g. age, phenotype severity), but to train a model able to predict individualized trajectories of HFMSE disease progression on the basis of the individual characteristics of the patient.

Following the suggestion that a composite and individualized model may improve the prognostic accuracy of disease progression [12, 13], we applied a machine learning approach using an XGBoost algorithm for regression. The advantage and peculiarity of this method compared to multivariate analysis is that it provides an estimate of the possible individual trajectory based on the baseline features, each of them assessed to establish their prognostic value. Moreover, unlike predictive models which rely on baseline features only, our approach is able to update the trajectory prediction at each visit time-point, thus capturing the dynamics of each exploratory variable over time.

The results of our analysis confirmed that age is an important prognostic factor but also showed that other variables may contribute to influence the progression of the disease. The analysis allowed to establish that HFMSE value at visit, i.e the first assessment of the two in a given interval, also appears to have a relevant impact on the prediction. Other variables, such as age at symptoms onset, as well as the HFMSE value recorded on the very first visit and BMI, also partially contributed to the prediction. Cobb’s angle and non-invasive ventilation were also very relevant, suggesting that increasing scoliosis and ventilatory status also contribute. In contrast, other variables such as gender and *SMN2* copies, did not appear to have a strong influence, as suggested by previous studies using multivariate analysis [5, 9].

These results should be interpreted with caution as the study was meant to be a proof of concept on a relatively small cohort and the results need to be validated in a larger cohort. The principal limitations of this study are that natural history data were drawn from a single center and that, even if there was a relatively large number of 6 month and 12 month follow-up intervals (>300 visits), the number of patients was much smaller (n = 30). Furthermore, it is known that the HFMSE is able to measure progression overtime but that can present floor/ceiling effect depending on age and functional status [15, 16, 19, 21, 22]. To address these concerns, additional work is in progress to establish external validity in separate datasets with a greater sample size. To address the issue of floor/ceiling effect in the prediction of the results, the choice of a predictive model whose underlying algorithm can introduce non-linear effects by partitioning the variables’ space into different sets, helps the predictive accuracy, as the model is not prone to extrapolation as it would be with linear or polynomial models.

In conclusion, our results suggest a possible role of this method that uses different criteria than those used in previous studies. Rather than providing a general rate of progression for a cohort or identify factors contributing to the progression through a multivariate analysis, the model can potentially provide more individualized trajectories. The application of this method to larger cohorts may allow to identify different classes of progression. The need to define more precise trajectories and predict patient outcome is crucial at the time when real world data from the commercially available new therapies are becoming increasingly available and there is the need to measure drug effect or potential treatment effect. This information may also be potentially used for clinical trial design to reduce variability and manage inclusion and criteria stratification.
